# Investigating Exogenous Tyrosine Supplements on the Responses of the Kale Plant to Salinity Stress

**DOI:** 10.1002/fsn3.70660

**Published:** 2025-07-20

**Authors:** Nezahat Turfan, İskender Khubalıyev, Kübra Tekşen, Ergin Murat Altuner

**Affiliations:** ^1^ Faculty of Science, Department of Biology Kastamonu University Kastamonu Türkiye

**Keywords:** antioxidants, nutrition, sustainability, tolerance

## Abstract

The study investigated the role of exogenous tyrosine (TYR) supplements in extending kale tolerance to NaCl stress at various concentrations (50, 100, and 200 mM). The salt stress was induced by irrigating the soil with salt water, and TYR was supplied through foliar spraying. The impact of TYR supplementation under NaCl stress was assessed by evaluating growth parameters, enzymatic and non‐enzymatic defense, oxidative stress markers, and mineral composition. The results revealed that TYR significantly increased the levels of β‐carotene, lycopene, anthocyanins, and polyphenols as well as the activities of polyphenol oxidase (PPO), catalase (CAT), and superoxide dismutase (SOD). TYR enhanced the activities of ascorbate peroxidase, CAT, and SOD under 200‐NaCl, increased PPO activity at all NaCl concentrations, and reduced the MDA content only at 200‐NaCl. The Mg, P, K, Ca, Na, and the ratio K/Na increased under 200‐NaCl, while Ca, Na, and Cl declined with lower NaCl. TYR raised Ca and Na levels at 100‐NaCl but decreased Na, Cl, and the Na/K ratio at 200‐NaCl. In conclusion, high NaCl levels suppressed Chl‐a, β‐carotene, lycopene, sucrose accumulation, and the activities of PPO, APX, CAT, and SOD, which led to reduced leaf, shoot, and root growth; however, these negative impacts were alleviated by TYR supplementation. The study suggests that to promote agricultural sustainability, it may be advisable to extend tolerance thresholds for moderately tolerant crops, enhance the tolerance of salt‐sensitive vegetables in saline regions, and incorporate exogenous TYR.

## Graphical abstract

Salt stress is one of the most important stresses that reduce plant yield. Tyrosine is useful in ameliorating the damage of salt stress. Kale is an important green leafy food source.

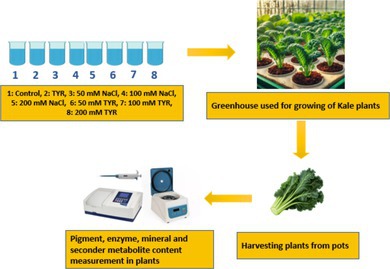

## 1 Introduction

Kale ( *Brassica oleracea var. acephala* ) is a nutrient‐rich vegetable with large, dark green leaves belonging to the Brassicaceae family. It has been reported to rank 15th among 47 vegetables recognized for their nutritional strength, thanks to its high levels of phytochemicals (Pascual et al. 2024). Kale, with its high content of vitamins (A, C and E), beneficial minerals (K, Ca, Mn, and Zn), prebiotic carbohydrates, dietary fiber, and low‐calorie profile, plays a vital role in managing and preventing conditions like obesity, hypertension, blood sugar imbalances, and digestive disorders. Further, it is also utilized in cancer treatment due to its secondary metabolites and antioxidant compounds (Pavlovic et al. 2019). Its cultivation began in the Mediterranean over 2000 years ago, and unlike other cruciferous vegetables, it is primarily cultivated for its leaves. Kale is an affordable food source for low‐income populations due to its lower cost compared to many other agricultural products. It is widely cultivated in Türkiye, particularly in the Black Sea region, including provinces such as Samsun, Ordu, Trabzon, and Rize, and serves as a valuable source of income for local communities (Balkaya and Yanmaz 2005). Its short growing season, adaptability to various climates, and ability to boost yields with organic and inorganic fertilizers make it an increasingly popular choice for cultivation. However, salt stress causes yield and quality loss in kale, as in other agricultural species. Kale is moderately sensitive to salinity, which limits its cultivation in arid and semi‐arid regions where soil salinity is a common challenge (Beacham et al. 2017; Pavlovic et al. 2019). Due to its moderate tolerance to salinity, kale emerges as a promising and robust candidate for research aimed at addressing the growing salinity challenges while also serving as a valuable food source to meet the increasing global food demands, with the world population projected to reach 9.7 billion by 2050 (United Nations 2024). Excessive levels of salinity lead to the accumulation of Na^+^ and Cl^−^ in the root zone, inhibiting water and mineral uptake by the roots and causing osmotic stress in both the soil solution and the cells, ultimately suppressing the development of roots, shoots, and leaves (Arif et al. 2019; Hasana and Miyake 2017). Indirect effects of salinity include the accumulation of ROS and MDA due to oxidative stress (Gomes et al. 2017), stomatal closure from disrupted osmotic adjustment, and inhibition of photosynthesis (Ciereszko 2018). Regulatory pigment degradation and perturbation of protein and enzyme structures (Turfan et al. 2024) are factors that can lead to developmental disorders and even plant death (Salachna et al. 2017). On the other hand, plant responses to salt stress vary based on genotype, growth stage, and the duration and severity of salinity (Khana et al. 2018). Recently, exogenous amino acid supplements have emerged as an innovative approach to enhance salt tolerance in plants, reduce salt‐induced damage, promote recovery, and improve both yield and nutritional quality. They are integral to the biosynthesis of biomolecules, including proteins, enzymes, vitamins, hormones, pigments, and secondary metabolites that directly or indirectly modulate plant productivity (Abdelkader et al. 2023). They can readily diffuse into leaf tissues following application, where they are either metabolized into nitrogenous compounds or utilized as a source of ATP, carbon, and nitrogen (Alasvandyaria and Mahdavia 2018). In this field, the ameliorative effects of amino acids such as proline (Hussain et al. 2018; Guo et al. 2022), glycine betaine (GB) (Khana et al. 2018; Sofy et al. 2020), and glutamic acid (Franzoni et al. 2022) have been demonstrated in species including *Brassica*, alfalfa, bean, and lettuce. All boost plant productivity in stressed and unstressed plants by promoting photosynthesis, pigment, and osmolyte synthesis (Hussain et al. 2018) and regulating water relations (Guo et al. 2022). Further, aromatic amino acids, phenylalanine (PHE), tryptophan (TRP), and tyrosine (TYR), in secondary metabolite‐rich species, contribute to biosynthesis via the shikimate pathway and activate non‐enzymatic and enzymatic antioxidant defenses under abiotic stress (Schenck et al. 2020). The beneficial effects of PHE, TRP, and TYR in elevating abiotic stress resilience have been substantiated in species including cucumber (Marium et al. 2021), spinach (Turfan et al. 2024), and arugula (Al‐Mohammad and Al‐Taey 2019).

Regarding TYR, its most distinctive feature compared to other amino acids is the hydroxyphenol group, which enables it to directly neutralize ROS and activate antioxidant enzymes. This group also underpins TYR's pivotal role in photosynthetic metabolism through two distinct mechanisms: by facilitating electron transport initiated by water splitting at Photosystem II, thereby mitigating chlorophyll degradation, and by serving as an integral structural component of the electron transport complexes embedded within the thylakoid membranes (Schenck et al. 2020). Additionally, TYR's capacity to perform ameliorative functions commonly attributed to other amino acids (Bakry et al. 2020; Atif et al. 2023) provides a unique advantage, thereby justifying its selection in this study. Therefore, elucidating the influence of TYR on salt tolerance mechanisms can provide valuable insights into developing sustainable strategies and resistant cultivars as well as for enhancing the tolerance thresholds of moderately tolerant genotypes such as kale. This knowledge may also help reduce the time required to improve tolerance in sensitive species. Previous studies have focused on determining the salt tolerance capacity of kale in comparison to other *Brassica* species (Salachna et al. 2017; Pavlovic et al. 2019; Pascual et al. 2024). Furthermore, the effects of salinity on salt responses in *Brassica* species, along with the impact of exogenous amino acid applications, particularly proline and glycine betaine, osmoprotective amino acids, have been investigated by Hussain et al. (2018) and Khana et al. (2018). Despite extensive documentation of TYR supplementation effects in crops such as wheat, peanut, aromatic species, and spinach, no studies in Türkiye have specifically addressed TYR's influence on enhancing the salt tolerance threshold of moderately salt‐tolerant kale cultivars. This gap highlights the urgent need for focused research in this area. In this study, it is hypothesized that exogenous TYR supplementation during the early growth stage may enhance kale's adaptation to salinity stress by activating multiple defense mechanisms, thereby improving its physiological resilience and biochemical stability under elevated salt stress. The primary objective of this study is to investigate the effects of TYR supplementation on the physiological and biochemical responses of kale. With its moderate salinity tolerance, it emerges as a promising and robust candidate for research on salt stress resilience. This may offer an innovative approach to evaluating TYR's role in enhancing kale's tolerance under varying salinity conditions and is expected to provide advanced insights beyond those offered by existing studies on other amino acids and biostimulants.

## 2 Materials and Methods

### 2.1 Cultivation Arrangements

**FIGURE 1:**
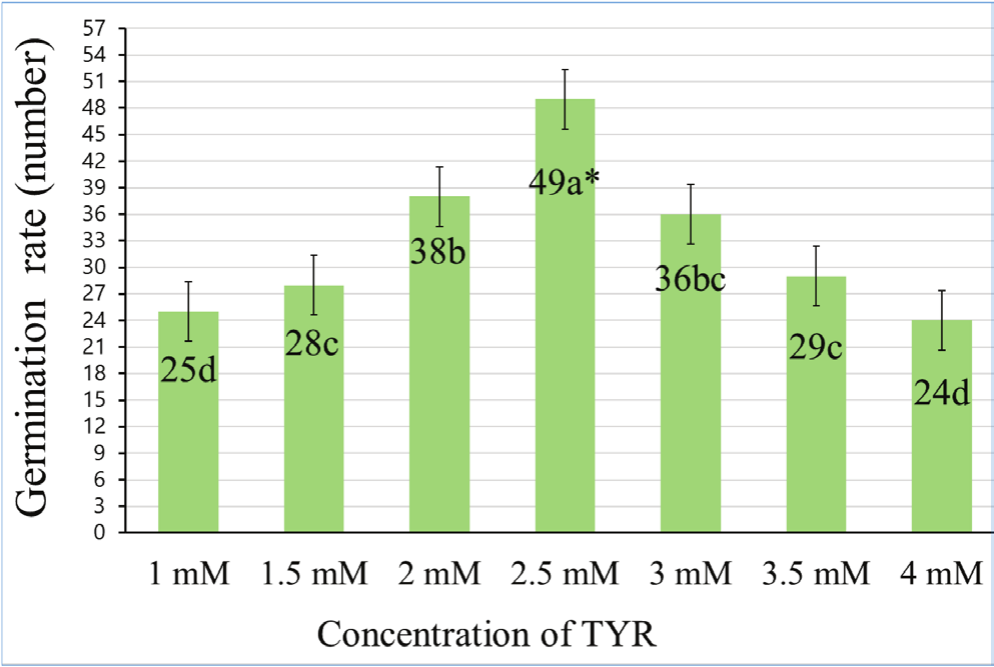
Defining the promotive dose of TYR in kale seedling germination. *Mean values (*n* = 3) in the same column for each trait in each group with the same lower‐case letter are not significantly different by Tukey's multiple range test at *p* ≤ 0.05. TYR, tyrosine.

The seeds were surface‐sterilized with a 5% sodium hypochlorite solution for 5 min and then cleaned with distilled water. A nutrient solution (Hoagland and Arnon 1950) was prepared with different concentrations of TYR (1, 1.5, 2, 2.5, 3, 3.5, and 4 mM). Solutions were transferred to beakers according to the given concentration order, and seeds were kept in these solutions for 4 h. Seeds were planted in petri dishes, 25 in each, and placed in a plant growth chamber at 22°C ± 2°C, 65% humidity, 16 h of light and 8 h of darkness. The dose of L‐tyrosine (TYR), which promotes growth in unstressed plants, was determined based on germination test results. As shown in Figure 1, seed germination counts ranged from 24 to 49, with the 2.5 mM dose promoting over 60% germination and supporting vigorous seedling development; thus, it was identified as the stimulating dose.

In the second stage of the experiment, sterilized seeds were planted in pots (16 × 17cm) filled with 3 L of peat and perlite mixture (3:1) as 5 seeds. The pots were grouped into eight categories: Control, TYR, 50‐NaCl, 100‐NaCl, 200‐NaCl, TYR‐50, TYR‐100, and TYR‐200 (Table 1).

**TABLE 1:** Treatments supplied in the study, concentrations, and abbreviations.

Application	Concentration	Abbreviation
1	Control	C
2	2.5 mM‐Tyrosine (Sigma‐Aldrich, CAS 35424–81‐8)	TYR
3	50 mM NaCl (Merck 106404.1000)	50‐NaCl
4	100 mM NaCl	100‐NaCl
5	200 mM NaCl	200‐ NaCl
6	2.5 mM Tyrosine −50 mM NaCl	TYR‐50
7	2.5 mM Tyrosine −100 mM NaCl	TYR‐100
8	2.5 mM Tyrosine −200 mM NaCl	TYR‐200

The seeds were fed only with Hoagland nutrient solution until the 4–5 leaf seedling stage. After this stage, three seedlings that showed good growth were selected for the application. Stress treatment involved watering the soil (125 mL) with NaCl solutions (50, 100, and 200 mM) dissolved in a nutrient solution, while TYR was applied by spraying 30–40 mL onto the leaves; in the control group, only the nutrient solution was sprayed. Applications were performed twice a week for 6 weeks. Pot experiments were conducted in a greenhouse at 24°C ± 2°C, with a relative humidity of 60% ± 5%. Seedlings were harvested in the seventh week and separated for morphological measurements and chemical analyses.

### 2.2 Morphological Measurements

While the root length (cm) was employed by considering the distance from the root of the seedling to the root tip, the shoot length (cm) was calculated by considering the distance from the root of the seedling to the shoot tip. The leaf length (cm) was measured by measuring the distance between the petiole and lamina. The fresh weight of a whole seedling and a leaf was weighed using precision scales.

### 2.3 Biochemical Analysis

The pigment analysis of leaves chlorophyll a (Chl‐a), chlorophyll b (Chl‐b), total chlorophyll (Chl), carotenoids, and lutein was achieved using the method outlined by Kukric et al. (2012). Examinations of β‐carotene and lycopene were conducted using the method described by Nagata and Yamashita (1992). A fresh tissue (1000 mg) was macerated with 20 mL of acetone‐hexane (4:6) at once, and then, the absorbance of the homogenate at 663, 645, 505, and 453 nm was recorded by a spectrophotometer at the same time.

The content of β‐carotene and lycopene was calculated using the following equations, and their concentrations were expressed as μg g^−1^.

$$\text{Lycopene}\;\left(\mathrm{mg}\;100\;{\mathrm{mL}}^{-1}\right)=-0.0458A_{663}+0.20{\text{4A}}_{645}+0.37{\text{2A}}_{505}-0.0806A_{453}$$

$$\beta -\text{carotene}\;\left(\mathrm{mg}\;100\;{\mathrm{mL}}^{-1}\right)=0.21{\text{6A}}_{663}-1.22A_{645}-0.30{\text{4A}}_{505}+0.45{\text{2A}}_{453}$$

where A: absorbance.

The total anthocyanin level was determined by the methods described by Mancinelli (1990). Firstly, 500 mg of fresh tissue was crushed with 3 mL of methanol‐HCl (1%) solution, and all homogenates were shaken for 48 h. After filtration of the extracts, the absorbance was taken at 530 nm. The anthocyanin concentration of samples was calculated with the given formula, utilizing an extinction coefficient of 31.6 M^−1^·cm^−1^.

$$\text{Anthocyanin}\;\left(\text{μmol}\;g^{-1}\right)=\left[\left(A_{530}-0.33\times A_{657}\right)/31.6\right)]\times \left(V/m\right)$$

m, amount of sample (g); V, volume of solution used (mL).

The soluble sugar content was examined using the anthrone reagent, following the method used previously by Turfan (2023). First, fresh samples were kept with 10 mL of 80% ethanol and then filtered. Anthrone reagent was poured into the mixture and incubated at 45°C–50°C for 15 min. The absorbance of the cooled samples was recorded at 630 nm, and the glucose content was measured using a glucose standard curve. The residue was filtered in pure water (+4°C) for 24 h. After joining the anthrone reagent to the filtrate, the absorbance was taken at 620 nm. The sucrose amount of the samples was measured using the standard curve prepared with sucrose monohydrate and given as mg/g^−1^.

Proline was assessed using the method of Bates et al. (1973). Fresh tissue (500 mg) was homogenized in 5 mL of 3% sulfosalicylic acid and centrifuged at 5000 rpm for 10 min. The reaction mixture, consisting of 2 mL homogenate, 2 mL acid ninhydrin, and 2 mL glacial acetic acid, was boiled at 100°C for 1 h. After cooling, 4 mL of toluene was added, and absorbance was measured at 520 nm using a spectrophotometer (UV‐160A, Shimadzu). The proline content was calculated from a standard curve and expressed in micrograms.

Polyphenol content was measured using Folin and Denis (1915). Fresh tissue (500 mg) was crushed with 80% methanol and filtered. A 500 mL sample was mixed with 2.5 mL of 10% Folin–Ciocalteu reagent and 2 mL of 7.5% Na_2_CO_3_ and then incubated in the dark for 2 h. Absorbance was measured at 760 nm, and polyphenol levels were quantified using a tannic acid standard curve (*R* ^2^: 0.988, *y* = 0.0128*x* − 0.0154).

Polyphenol oxidase (PPO) activity was quantified spectrophotometrically following the method depicted by Galeazzi et al. (1981), which is based on the oxidation of catechol to 1,2‐benzoquinone. Activity assessment was determined with a mixture of 900 μL of catechol dissolved in sodium phosphate buffer (0.1 M, pH 7.0) and 100 mL of enzyme extract. The absorbance of supernatants was taken at 420 nm at two‐minute intervals, and PPO activity was computed as the slope of the linear portion of the resulting activity curve. One unit of PPO (U mg^−1^ Protein) activity was defined as the amount of enzyme causing an increase in absorbance of 0.001 per minute in a 1 mL reaction mixture.

### 2.4 Estimation of Oxidative Stress

Fresh tissue (500 mg) was homogenized in 5 mL of 3% TCA in an ice bath and centrifuged at 12,000 rpm for 15 min at 4°C. The homogenate was then divided for the measurement of MDA (malondialdehyde) and H_2_O_2_ (hydrogen peroxide). For MDA, 500 mL of homogenate was mixed with 500 mL of 20% TCA and 0.5% TBA, then boiled at 100°C for 60 min. After cooling, the mixture was centrifuged at 12,000 rpm for 10 min. Absorbance was measured at 532 and 600 nm using a UV–Vis spectrophotometer (UV‐160A, Shimadzu). MDA concentration was calculated in μmol g^−1^ fresh weight using an extinction coefficient of 155 mM (Lutts et al. 1996). For H_2_O_2_, 500 mL of homogenate was mixed with 500 mL of phosphate buffer (pH 7) and 1 mL of 1 M potassium iodide. Absorbance was measured at 355 nm, and H_2_O_2_ content was quantified using Velikova et al.'s (2000) method. For the extraction of the antioxidant enzyme, a sodium phosphate buffer solution (50 mM, pH 7.6) was used. The leaf tissues (500 mg) were coalesced in a 5 mL buffer containing 0.1 mM Na‐EDTA (ethylenediamine tetra‐acetic acid disodium salt). All homogenates were centrifuged for 15 min at 15000 rpm at 4°C, and the homogenate was evaluated for the measurement of the activities of antioxidant enzymes such as ascorbate peroxidase (APX), catalase (CAT), peroxidase (POD), and superoxide dismutase (SOD) (Zhang et al. 2006).

The activity of APX assessment was performed with a reaction amalgam prepared with 50 mM phosphate buffer (pH 7.0), 0.55 mM ascorbic acid, 50 mL of enzyme extract, and 0.1 mM H_2_O_2_. The absorbance of the mixture was taken for 20 min at 290 nm. The CAT activity of samples was assayed with a mixture of 100 mM sodium phosphate buffer (pH 7.0), 30 mM H_2_O_2_, and 100 μL of crude extract in a total volume of 3.0 mL. CAT activity was expressed as the amount of enzyme that caused an absorbance change of 0.001 per minute. The activity of POD was quantified with a mixture consisting of potassium phosphate buffer (50 mM, pH 7.0), 19.4 μL of H_2_O_2_ (35%), 50 μL of extract, and guaiacol (33 μL). The absorbance was taken at 470 nm, considering the change in absorbance for 1 min. POD activity was given as U μL^−1^·min^−1^. SOD activity was measured by inhibition of the photochemical reduction of nitroblue tetrazolium (NBT). A 3 mL reaction mixture containing crude extract, phosphate buffer (pH 7.0), riboflavin, methionine, NBT, and EDTA was prepared. A lamp irradiated the test tubes with white fluorescence for 15 min. The absorbance of solutions was noted at 560 nm. One unit (U) of SOD activity was expressed as a U mg^−1^ protein.

Some soil samples were collected before arranging experiments to assay elemental quantification (Mg, P, S, K, Ca, Na, and Cl). The collected fresh leaves and soil samples were dried at 65°C in an oven and powdered. The powdered samples were used to assess the elemental analysis at Kastamonu University, Central Research Laboratory, using the ICP‐OES (SpectroBlue II) device. Each sample was analyzed in triplicate. The pH values of the soil samples were determined using a digital pH meter.

### 2.5 Statistical Analysis

Following the results of MANOVAs, Tukey's honestly significant difference (HSD) test (*α* = 0.05) was used for post hoc analysis to determine which group pairs differ significantly. The Tukey HSD was performed using R Studio, and biplot graphs in principal component analysis (PCA) were conducted to demonstrate relationships between variables and observations in a lower‐dimensional space.

## 3 Results and Discussion

### 3.1 The Growth Rate of Kale Seedlings

Salinity inhibits cell proliferation in meristematic zones of shoots and roots (Hasana and Miyake 2017), causes nutritional deficiencies by suppressing photosynthesis, and induces oxidative stress, damaging pigments and enzymes, impairing plant growth (Gomes et al. 2017). In this study, the impacts of foliar TYR supplement on the growth rate traits of kale are shown in Table 2. The difference among the applications regarding growth rate parameters was significant at the *p* < 0.01 level. The result displayed that plants stressed with NaCl, particularly at 200‐NaCl, exhibited the lowest values for shoot, root, and leaf length, fresh weight of the whole plant and leaf, and leaf number, compared to the control. In contrast, TYR supplementation to unstressed seedlings had a promotive effect on these parameters. Compared to the control, the most pronounced reductions in shoot and root lengths were observed under 200‐NaCl (by 88% and 56%) (Table 2). Finally, TYR supplementation to NaCl‐stressed plants considerably improved growth rate parameters, though still lower than the control, with the greatest improvement in the 50‐NaCl (Table 2).

**TABLE 2:** Variation of shoot, root and leaf growth rate traits of kale seedlings.

Group	Shoot length (cm)	Root length (cm)	FW of seedling (g)	Leaf length (cm)	Blade length (cm)	Blade width (cm)	FW of leave (g)	Leaf number
Control	15.33 ± 0.24a***	10.12 ± 0.16ab***	35.33 ± 0.16b***	10.01 ± 0.13a***	9.21 ± 0.12a***	5.12 ± 0.18a***	3.04 ± 0.06b***	10.50 ± 0.53ab***
TYR	16.75 ± 0.16a***	12.11 ± 0.13a***	40.54 ± 0.45a***	10.18 ± 0.17a***	9.77 ± 0.20a***	5.17 ± 0.20a***	4.87 ± 0.04a***	11.80 ± 0.79a***
50‐NaCl	12.14 ± 0.19b***	9.74 ± 0.12b***	24.04 ± 0.18 cd***	5.84 ± 0.23bc***	6.04 ± 0.25b***	3.04 ± 0.15bc***	2.15 ± 0.07bc***	10.00 ± 0.82b***
100‐NaCl	9.84 ± 0.14c***	7.34 ± 0.14c***	15.89 ± 0.09e***	5.51 ± 0.22c***	5.39 ± 0.28c***	2.85 ± 0.13c***	1.64 ± 0.07d***	9.20 ± 0.42b***
200‐NaCl	8.61 ± 0.09d***	6.27 ± 0.12d***	12.51 ± 0.12f***	5.21 ± 0.30d***	5.18 ± 0.32c***	2.79 ± 0.13c***	1.42 ± 0.09e***	9.00 ± 0.47c***
TYR‐50	13.97 ± 0.15ab***	9.69 ± 0.10b***	26.77 ± 0.13b***	7.12 ± 0.21b***	8.29 ± 0.24ab***	3.94 ± 0.24b***	2.96 ± 0.07b***	10.90 ± 0.88ab***
TYR‐100	10.76 ± 0.16bc***	8.89 ± 0.16bc***	23.12 ± 0.14d***	7.12 ± 0.25b***	6.78 ± 0.14b***	3.34 ± 0.23bb***	2.38 ± 0.09bc***	9.40 ± 0.70b***
TYR‐200	9.98 ± 0.17c***	6.34 ± 0.18d***	16.98 ± 0.12e***	6.21 ± 0.16bc***	6.26 ± 0.17b***	3.22 ± 0.20c***	1.91 ± 0.06c***	9.60 ± 0.70b***
*F*	307.6	212.8	2275	83.33	63.27	26.79	283.9	19.83

*Note:* Mean values (*n* = 10) in the same column for each trait in each group with the same lower‐case letter are not significantly different by Tukey's multiple range test at *p* ≤ 0.05. ***p < 0.001, TYR, Tyrosine.

Salt‐induced retardation of leaf, shoot, and root growth in leafy vegetables such as lettuce, kale, and spinach was demonstrated by Franzoni et al. (2022), Pavlovic et al. (2019), and Turfan (2023), whereas the mitigating effects of foliar‐applied amino acids, such as PHE, PRO, and TRP, were highlighted in studies by Marium et al. (2021), Hussain et al. (2018), Khana et al. (2018), and Turfan et al. (2024) in cucumber, *Brassica*, and spinach. The decrease in growth rate observed with NaCl exposure may be attributed to impaired water and mineral uptake due to root cell damage (Arif et al. 2019), decreased carbon assimilation resulting from stomatal closure (Beacham et al. 2017), and inhibited nitrogen utilization (Seyedi et al. 2024). Moreover, while the promotive effect of TYR on growth‐related traits in NaCl‐stressed spinach and peanuts was reported by Turfan (2023) and Bakry et al. (2020), Al‐Mohammad and Al‐Taey (2019), Feduraev et al. (2020), and Helal and Ibrahim (2019) observed similar effects of TYR on quinoa, wheat, and *Hibiscus sabdariffa* under non‐stress conditions. The improvement of the growth rate by TYR may be attributed to its roles in promoting pigment synthesis, secondary metabolite production, and osmolyte accumulation (Abdallah and El‐Bassiouny 2016) as well as its ability to inhibit oxidative stress by activating key enzymes (Guo et al. 2022).

### 3.2 Variation of Pigments and Secondary Metabolites Contents

**FIGURE 2:**
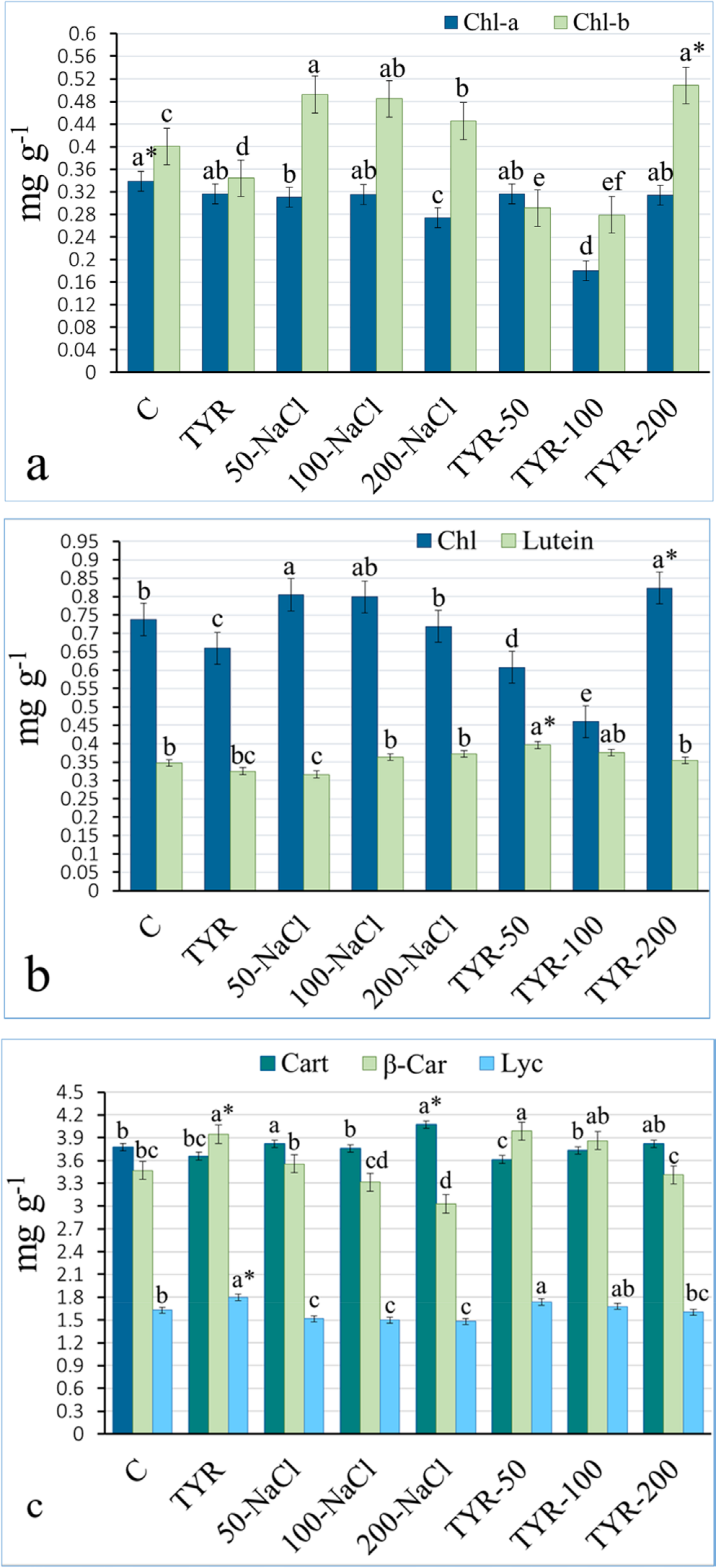
Variation of (a) chlorophyll a (Chl‐a), chlorophyll b (Chl‐b), (b) total chlorophyll (Chl), lutein, (c) carotenoid, β‐carotene (β‐car), and lycopene (Lyc) in the kale leaves. *Mean values (*n* = 3) in the same column for each trait in each group with the same lower‐case letter are not significantly different by Tukey's multiple range test at *p* ≤ 0.05. TYR, tyrosine.

Numerous researchers have reported that chlorophyll pigments (Chl‐a, Chl‐b, and total Chl), particularly Chl‐a, undergo rapid degradation under salt stress conditions, leading to observable symptoms such as color loss or chlorosis in plants (Gomes et al. 2017; Zhu et al. 2017), consistent with our results. As summarized in Figure 2a,b, TYR supplementation in unstressed plants resulted in a reduction of chlorophyll content compared to the control, which may be explained by a dilution effect caused by the expansion of leaf surface area (Turfan 2023). Furthermore, Chl‐a content declined considerably in both NaCl‐stressed plants and TYR supplemented to NaCl groups, with the sharpest reductions recorded in TYR‐100 (47%) and 200‐NaCl (19%) stress. While Chl‐b accumulation increased inversely with the three NaCl doses (22%, 21%, and 11%), Chl peaked at 50 and 100‐NaCl (Figure 2a,b). The increased levels of Chl‐b and Chl under a saline environment are considered an adaptive mechanism aimed at maximizing light harvesting, thereby enhancing photosynthetic efficiency. Besides capturing and transferring energy, chlorophylls also help protect thylakoid‐embedded photosystems from oxidative stress due to their antioxidant properties (Cazzaniga et al. 2016). Higher NaCl concentrations have been shown to significantly reduce Chl‐a, Chl‐b, and total chlorophyll levels in *Brassica* compared to the control (Khana et al. 2018), with the decline in Chl‐a consistent with the present study, whereas the changes in Chl‐b and total Chl content were contrary. Salachna et al. (2017) observed a progressive decline in total chlorophyll content in curly kale under different NaCl concentrations, which intensified with prolonged salt exposure. Furthermore, the Chl‐promoting impact of TYR in unstressed *Salvia farinacea* leaves, as reported by Nahed and Balbaa (2007), aligned with the Chl enrichment observed in kale. In another study, Abdallah and El‐Bassiouny (2016) recorded a dose‐dependent increase in Chl‐b and Chl in quinoa leaves under salt stress with increasing TYR concentrations, which is consistent with the findings of the present study. Although high alkalinity led to a reduction in chlorophyll levels in spinach, TYR supplementation under alkaline stress not only mitigated this decline but also promoted chlorophyll accumulation, resulting in a notable increase even in stressed plants (Turfan 2023). Fluctuations in chlorophyll accumulation may be attributed to genotypes' responses to salt stress, the particular metabolic pathways in which TYR is directly involved, and variations in its interactions with salt ions.

Carotenoids are photoreceptors embedded in the thylakoid membrane, primarily present as lutein, β‐carotene, and other forms, often overlain by chlorophylls (Maoka 2020). Under stress conditions, they protect chlorophyll and thylakoid membranes from photooxidative damage through photoprotective mechanisms and by quenching excess harvested energy (Cazzaniga et al. 2016). Regarding carotenoids (total carotenoid, lycopene, β‐carotene, and lutein), variations in leaves are given in Figure 2c. A marked increase of 14.5% in β‐carotene and 10.4% in lycopene occurred in the unstressed group upon TYR supplementation compared to the control, whereas a decline was noted in total carotenoids and lutein levels. Increasing NaCl doses led to a reduction in β‐carotene and lycopene content, accompanied by a slight increment in lutein content. Further, TYR supplementation in NaCl‐stressed groups enriched β‐carotene, lycopene, and lutein levels at 50 and 100 mM but lowered total carotenoids at these concentrations; at 200‐NaCl, TYR supplementation led to a partial increase in total carotenoids and lutein (Figure 2c). As noted by Pascual et al. (2024), the observed decrease in β‐carotene and lycopene at 200‐NaCl is considered a coping strategy against salt‐induced oxidative stress in leaves. The enhancing effect of TYR on the synthesis of β‐carotene and lycopene through the activation of enzymes responsible for their biosynthesis was demonstrated in peanuts by Bakry et al. (2020). Similarly, Nahed and Balbaa (2007) observed an increase in total carotenoid content in *Salvia farinacea* with TYR alone, while Abdallah and El‐Bassiouny (2016) reported an elevation in carotenoid levels in quinoa grown under saline conditions following TYR supplementation. The decrease in lycopene and β‐carotene, along with the increase in lutein at 100 and 200‐NaCl, is consistent with the changes in carotenoid and lutein levels observed in spinach under salt stress (Turfan et al. 2024). Fluctuations observed in carotenoid content may be due to the differential salt tolerance characteristics of various carotenoid types, as well as changes in kale's physiological responses to salinity (Maoka 2020). Additionally, Gomes et al. (2017) and Zhu et al. (2017) indicate that carotenoid diversity and ratios in plant tissues depend on light conditions, organ maturity, and plant species. Furthermore, TYR, as a photoreceptor amino acid, modulates phosphorylation under saline stress, influencing enzyme activation in carotenoid biosynthesis and regulating carotenoid content and diversity (Schenck et al. 2020).

### 3.3 Variation of Secondary Metabolite Contents

**FIGURE 3:**
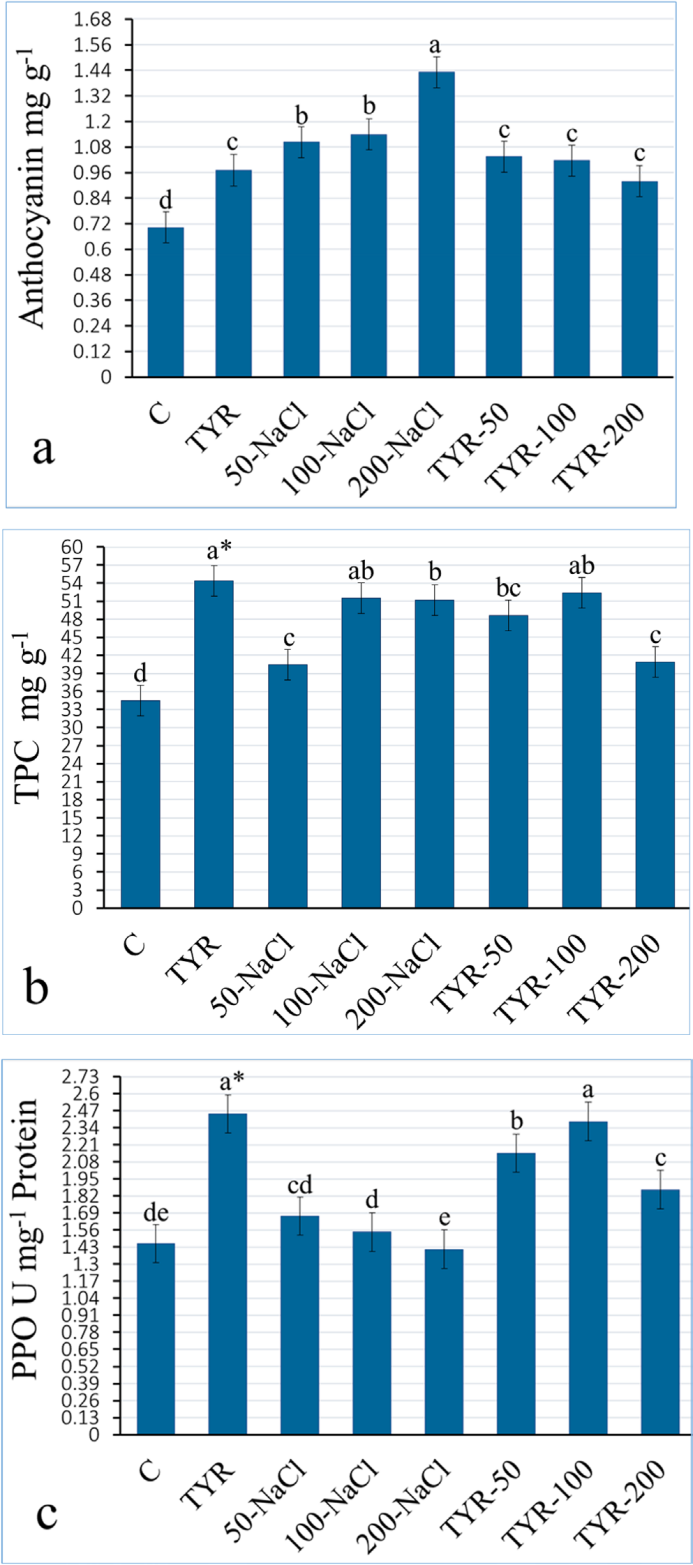
Variation of (a) anthocyanin, (b) total polyphenol (TPC), and (c) polyphenol oxidase (PPO) activity in the kale leaves. *Mean values (*n* = 3) in the same column for each trait in each group with the same lower‐case letter are not significantly different by Tukey's multiple range test at *p* ≤ 0.05. TYR, tyrosine.

In kale leaves, anthocyanin content ranged from 0.70 to 1.43 mg, showing an increase compared to the control under TYR, NaCl, and their combinations. The highest levels were achieved with 100 (62%) and 200‐NaCl groups (104%) (Figure 3a). The total phenolic content (TPC) of leaves ranged from 34.47 to 54.43 mg (control‐TYR). Compared to the control, the highest TPC was observed in the 100‐NaCl within the salt‐treated plants, and similarly, the highest value was recorded in the 100‐NaCl in the TYR‐supplemented stressed plants (Figure 3b). PPO activity was lowest in the 200‐NaCl compared to the control, with a substantial increase observed in the TYR (68%) and TYR‐100 (64%) groups (Figure 3c). A possible explanation for the accumulation of anthocyanin and TPC in kale leaves under NaCl stress is that these species are rich in secondary metabolites, which may act as protective agents, strengthening epidermal tissues against pathogen attacks (Beacham et al. 2017) and mitigating salt‐induced damage (Khana et al. 2018). Samec et al. (2021), in their study on phytochemical diversity and concentrations in various *Brassica* cultivars during exposure to NaCl (50–100 mM), reported TPC levels in unstressed and stressed seedlings ranging from 19 to 26.14 mg and 18.83 to 30.50 mg, respectively. Additionally, the highest TPC content was observed in the 100‐NaCl in kale, while the 50‐NaCl group yielded the highest levels in other *Brassica*. The elevation of anthocyanin and TPC levels, as well as PPO activity in kale leaves following TYR treatment, may be attributed to the activation of secondary metabolite biosynthetic pathways by TYR (Jain and Bhatla 2018). Anthocyanins and polyphenols are synthesized from aromatic amino acids via the shikimate pathway; thus, their levels in plant tissues may be closely associated with TYR metabolism (Feduraev et al. 2020). Owing to their polyhydroxylated structures, they effectively scavenge ROS generated under salinity stress (Zhu et al. 2017). Likewise, the stimulative effect of TYR administration on anthocyanin and polyphenol accumulation in both salt‐stressed and non‐stressed plants has also been outlined for maize (Atif et al. 2023) and arugula (Arif et al. 2019). Moreover, PPO, which typically functions as a key enzyme activating plant defense mechanisms, is responsible for catalyzing the oxidation of TPC (Jain and Bhatla 2018). TYR, serving as a precursor amino acid in the biosynthesis of phenolic compounds, can indirectly enhance PPO activity by modulating the availability of its substrates. High salt levels can reduce PPO activity by limiting the availability of its substrate, as observed at 200‐NaCl in this study (Figure 3c). Bakry et al. (2020) found that TYR supplementation in peanut seedlings activated secondary metabolic pathways. Turfan (2023) demonstrated that TYR spraying on Li‐stressed spinach increased PAL activity, thereby enhancing TPC and anthocyanin content in stressed seedlings.

**FIGURE 4:**
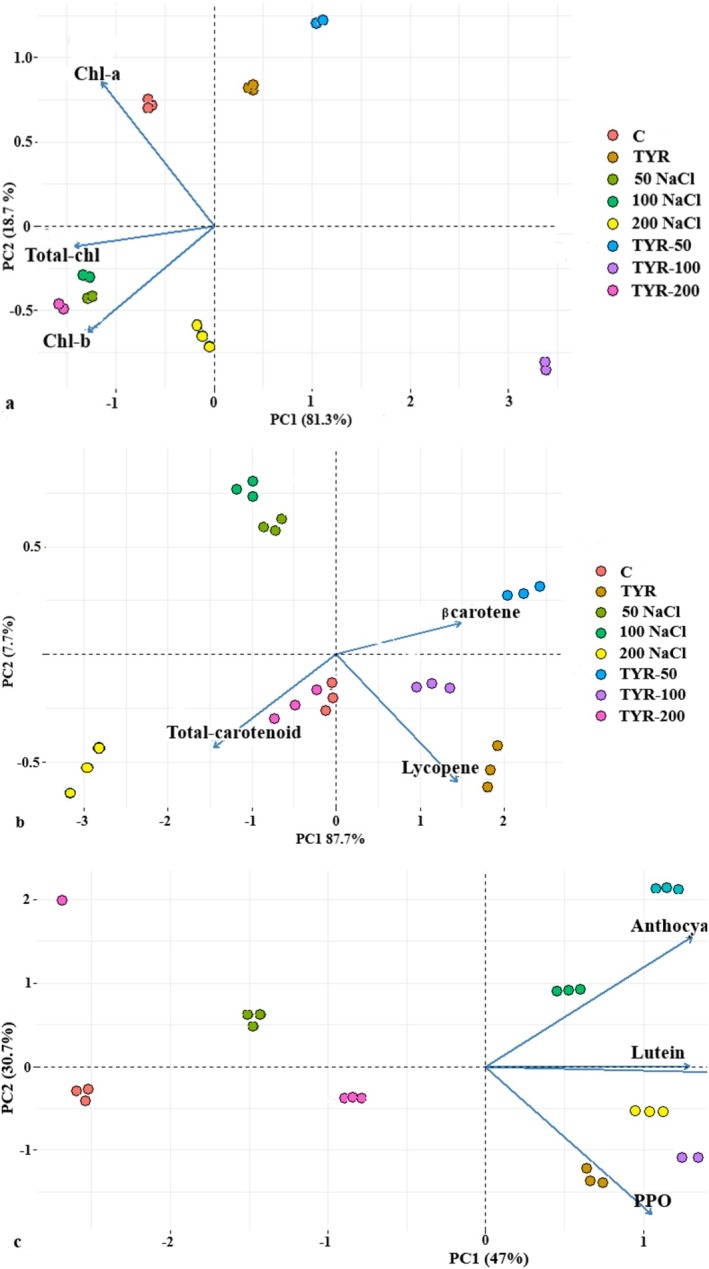
PCA biplot analysis for the amount of (a) Chl‐a, Chl‐b, total Chl. (b) carotenoid, lycopene, β‐carotene. (c) lutein, anthocyanin, polyphenol, and the activity of polyphenol oxidase (PPO).

The biplot graph generated by Principal Component Analysis (PCA) displays both the score and loading plots for the different experimental conditions and measured variables. The spatial distribution of treatment groups reflects their compositional differences; groups located further apart indicate a greater degree of dissimilarity in their content. The eigenvectors in the biplot represent the direction and magnitude of variable contributions to the principal components. The angle between these vectors provides insight into the correlation between variables. Vectors forming acute angles, such as POD, CAT, APX, SOD, and proline, suggest a strong positive correlation between these antioxidant enzymes and osmolytes. This indicates that an increase in one of these parameters is likely associated with an increase in the others, implying a coordinated stress response. In contrast, vectors forming right angles (e.g., sucrose and proline) imply no significant correlation between those variables, reflecting independent behavior under the given conditions. Moreover, obtuse angles (greater than 90°), such as those between β‐carotene and total carotenoids, indicate a negative correlation, suggesting that as the content of one increases, the other tends to decrease. The primary differences among the groups were primarily captured along PC (Principal Component) 1. In the PCA of total chlorophyll, chlorophyll a, and chlorophyll b content, PC1 and PC2 accounted for 81.3% and 18.7% of the total variation (Figure 4a).

This means that PC1 captured the majority of the variability in the data related to these variables. Similarly, in the analysis of total carotenoid, beta‐carotene, and lycopene content, PC1 and PC2 explained 87.7% and 7.7% of the total variation (Figure 4b). Furthermore, when considering differences in H_2_O_2_, MDA, sucrose, glucose, POD, CAT, APX, SOD, and proline, PC1 and PC2 explained 73.1% and 15.8% of the variance (Figure 7). Similarly, in the analysis of elements such as lutein and anthocyanin, PC1 and PC2 accounted for 62.7% and 20.5% of the total variance (Figure 4c).

**FIGURE 5:**
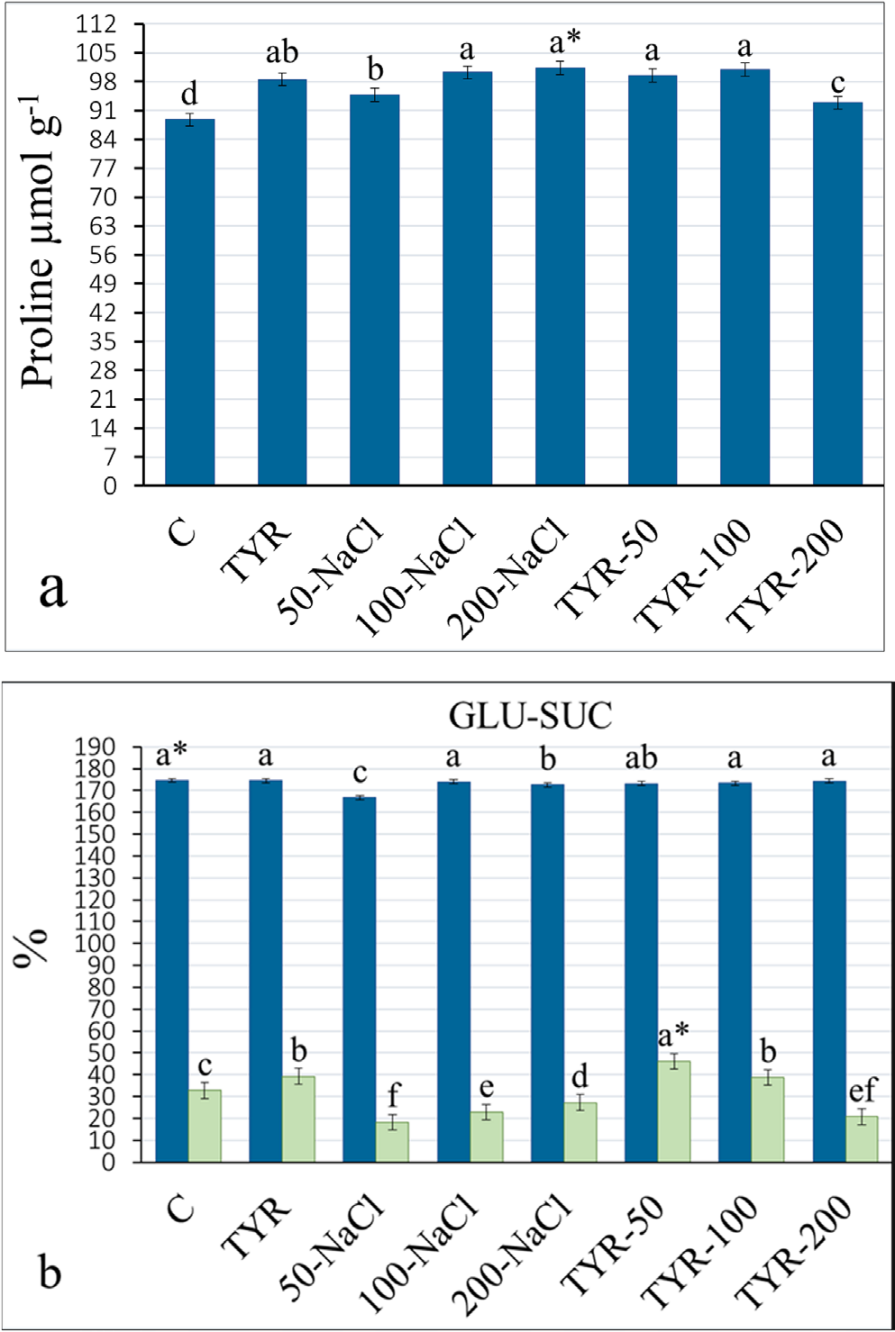
Variation of (a) proline, (b) glucose (GLU), and sucrose (SUC) content of kale leaves. *Mean values (*n* = 3) in the same column for each trait in each group with the same lower‐case letter are not significantly different by Tukey's multiple range test at *p* ≤ 0.05. TYR, tyrosine.

One of the primary cellular responses to salt stress is the accumulation of compatible solutes, such as proline, glucose, and sucrose (Hussain et al. 2018), which play crucial roles in maintaining cellular water balance, protecting membrane integrity and cellular components, and alleviating oxidative damage by scavenging reactive oxygen species (Abdelkader et al. 2023). Proline accumulation was lowest in the control, with 14% and 11% rises observed under 200‐ and 100‐NaCl stress. TYR supplements alone led to an 11% enhancement compared to the control. Furthermore, TYR supplementation in NaCl‐stressed plants promoted proline levels, reaching the highest elevation (13.7%) at 200‐NaCl (Figure 5a). Supporting the outcomes of this study, Gomes et al. (2017) reported a significant proline accumulation in *Salvia auricula* leaves subjected to 150‐NaCl. A similar trend in proline accumulation under saline conditions was shown by Abdallah and El‐Bassiouny (2016) in quinoa following TYR supplementation. Similarly, Alfosea‐Simon et al. (2020) demonstrated in tomatoes that the inhibitory effect of salt stress on proline synthesis was removed by tyrosine supplementation. Proline accumulation under salt stress in *Brassica* cultivars was further promoted by proline and glycine betaine supplementation, as verified by Hussain et al. (2018) and Khana et al. (2018). In this study, it was thought that TYR may play a role in regulating proline content in kale plants by potentially inducing its de novo synthesis, inhibiting degradation, or facilitating its conversion into soluble sugars and secondary metabolites (Jain and Bhatla 2018).

In the samples, glucose (GLU) content (%) decreased with increasing NaCl concentrations; however, this decrease was only statistically significant in the 50‐ and 200‐NaCl (Figure 5b). Further, sucrose (SUC) levels decreased by 44.37% and 30.26% in the 50‐NaCl and 100‐NaCl, respectively. A study by Alfosea‐Simon et al. (2020) found that TYR supplements to tomatoes augmented GLU and SUC levels, in contrast to the control; however, while the results of this study align with the sucrose data, they contradict the GLU values. TYR supplementation in the unstressed plants resulted in a 19.74% increase. Moreover, TYR supplementation in the NaCl‐stressed group increased SUC in the 50‐NaCl (40.76%) and 100‐NaCl (18.27%), while reducing it by 36.6% in the 200‐NaCl (Figure 5a,b). El‐Sherbeny and Teixeira Da Silva (2013) reported a higher accumulation of total soluble sugars in beet leaves treated with TYR at concentrations of 100–400 mg (17%–24%) compared to the control (16.20%), attributing this increase to the carbon sources supplied by TYR. L‐TYR contributes to both protein synthesis and carbon metabolism, especially under nutrient‐deficient conditions, by serving as a precursor for pigments and secondary metabolites. In harmony with this study, Bakry et al. (2020) in peanuts and Atif et al. (2023) in maize reported that TYR application at various concentrations increased total soluble carbohydrate content in peanut plants compared to the control. Seyedi et al. (2024) investigated the impact of amino acid mixtures on Dracocephalum growth under salt stress, finding that amino acid supplementation increased soluble sugar levels, which likely aids osmotic adjustment and stress tolerance. The decrease in GLU and SUC under salt stress was linked to their use as energy and carbon sources (Ciereszko 2018), while the increase in SUC content with TYR supplementation was associated with enhanced photosynthetic pigment levels, thereby stimulating photosynthetic gain (Nahed and Balbaa 2007; Jain and Bhatla 2018).

**FIGURE 6:**
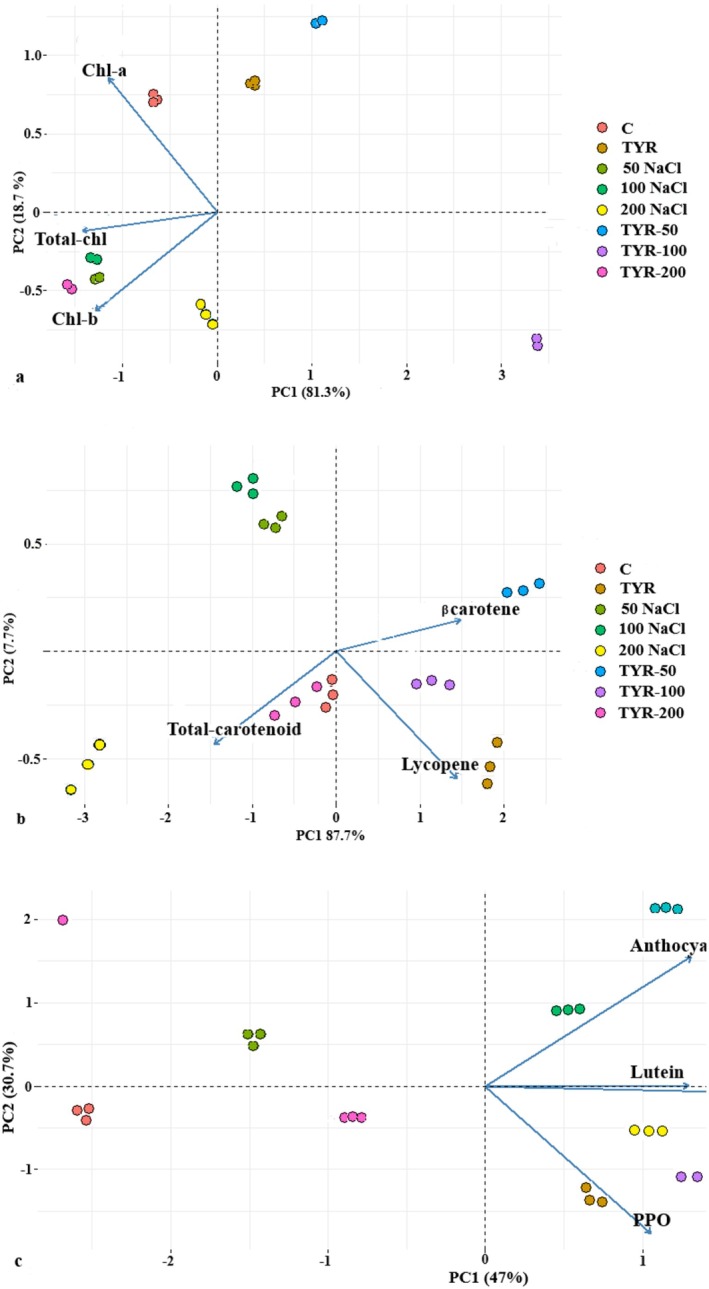
Variation of (a) malondialdehyde (MDA), (b) hydrogen peroxide (H_2_O_2_) concentration, and activity of (c) APX, CAT, POD, and (d) SOD in the kale leaves.*Mean values (*n* = 3) in the same column for each trait in each group with the same lower‐case letter are not significantly different by Tukey's multiple range test at *p* ≤ 0.05. TYR, tyrosine.

**FIGURE 7:**
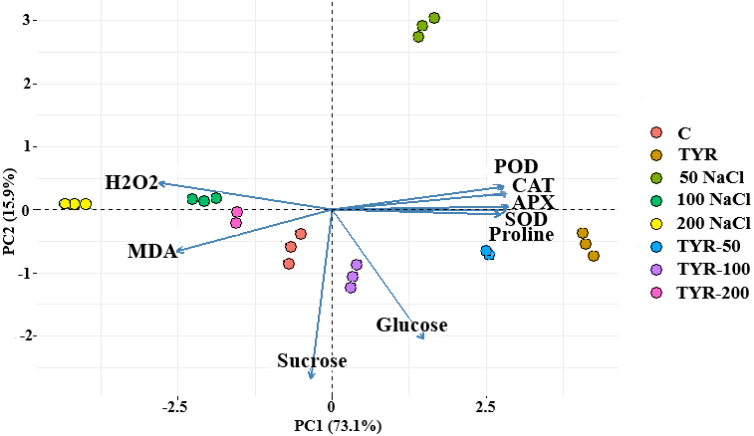
PCA biplot analysis for proline, glucose, sucrose, MDA, and H_2_O_2_, concentrations, and the activity of APX, CAT, POD, and SOD. C, control; TYR, tyrosine.

As a result of cellular metabolism, malondialdehyde (MDA) and ROS, including H_2_O_2_, superoxide anion, hydroxyl radical, and singlet oxygen, are generated. However, under stress conditions, their excessive accumulation can overwhelm the plant's tolerance threshold, adversely affecting its vitality and productivity (Turfan et al. 2024). As depicted in Figure 6a,b, individual applications of NaCl or TYR led to reduced MDA content compared to the control; however, their combined application under saline conditions resulted in a notable, concentration‐dependent increase in MDA levels by 3% and 11.4% under 50‐NaCl and 100‐NaCl, suggesting a potential interaction between TYR and salt stress in modulating lipid peroxidation. Furthermore, a 3.6% partial reduction in MDA content was observed with TYR supplementation under 200‐NaCl. H_2_O_2_ content was greatest in the control (195.03 μmol) and lowest in the TYR (14.6%) and TYR‐200 (19.7%) (Figure 6b). In comparison to the control, the lessening MDA and H_2_O_2_ concentrations with NaCl doses, particularly in 50‐NaCl (25.81%), were attributed to the elevated proline, anthocyanin, and lutein levels (Figure 6b). When considering differences in proline, GLU, SUC, MDA, H_2_O_2_, APX, CAT, POD, and SOD levels, PC1 and PC2 explained 73.1% and 15.8% of the variance (Figure 7). Under stress conditions, these compounds may serve as antioxidant agents by attenuating lipid peroxidation (Turfan et al. 2024) and neutralizing ROS induced by stress (Hussain et al. 2018). However, under salt stress conditions, as reported by Pavlovic et al. (2019) and Samec et al. (2021), tolerant cultivars actively restrict the accumulation of MDA and H_2_O_2_ through the use of compatible solutes, secondary metabolites, and antioxidant enzymes, thereby enhancing their oxidative stress tolerance. Aligned with this study, Turfan et al. (2024) reported that 200‐NaCl induced elevations in MDA and H_2_O_2_ levels in spinach; however, tryptophan application effectively lessened these increases by activating antioxidant enzymes, enhancing osmolyte accumulation, and directly scavenging ROS. A similar trend was observed in salt‐stressed *Brassica* species supplemented with proline and glycine betaine, as reported by Hussain et al. (2018), and also achieved in maize (Atif et al. 2023) through TYR application. The increase in MDA observed with TYR supplementation in the 50‐ and 100‐NaCl (39% and 43.88%, respectively) may be linked to the toxic effect of TYR in moderate‐tolerant cultivars (Jain and Bhatla 2018).

Echoing previous findings, fluctuations in MDA and H_2_O_2_ levels may result from the modulation of antioxidant enzyme activities, indicating that plants, especially stress‐tolerant varieties, activate enzymatic defense mechanisms to withstand salinity stress (Bakry et al. 2020). Despite an increase in APX activity under 50 and 100‐NaCl stress, the highest activity was noted in the TYR‐200 compared to the control (Figure 6c). CAT and SOD enzyme activities were lowest in the unstressed plants, with CAT reaching peak activity in the TYR‐200 and SOD exhibiting its highest activity under 200‐NaCl (Figure 6a,d). Also, a considerable inhibition of POD activity was recorded with the 200‐NaCl, followed by TYR‐100 (Figure 6c,d). These results suggest that while 200‐NaCl suppresses the activities of key antioxidant enzymes in kale, exogenous tyrosine supplementation can enhance enzymatic defenses, thereby contributing to improved salt stress tolerance in this species. Elevated activities of APX, CAT, POD, and SOD under moderate salinity were observed by Jain and Bhatla (2018) in sunflower and Turfan (2023) in spinach, corroborating our observations. APX and CAT reduce H_2_O_2_ accumulation by converting it to water, while POD and SOD contribute to the reduction of other reactive oxygen species (Hussain et al. 2018). Besides, the promotive effect of amino acids on enzyme activation during salt stress has been highlighted by Abdallah and El‐Bassiouny (2016) and Turfan et al. (2024). The highest activities of APX, CAT, POD, and SOD in seedlings stressed with 200‐NaCl and supplemented with TYR were linked to a specific interaction between TYR and salt concentrations (Jain and Bhatla 2018). Additionally, exogenous TYR supplements are effective with a higher salt dose, which can be explained by the studied cultivar that may resist low‐ and medium‐salt stress (Beacham et al. 2017). Apart from an increment in proline, anthocyanin, phenolic compounds (Feduraev et al. 2020), and enzyme activities responsible for secondary pathways may contribute to the elevation of APX, CAT, POD, and SOD activities (Schenck et al. 2020).

### 3.4 Variation of Mineral Content in Kale Seedlings

Although K^+^, Ca^2+^, Mg^2+^, and P are essential for plant growth and development, their excessive accumulation in plant tissues can induce ion toxicity. To avoid the potential threats of ion toxicity, plants accumulate these ions into vacuoles; however, when vacuolar capacity is exceeded, the ions begin to accumulate in the cytosol (Hasana and Miyake 2017). In this study, the alteration of Na, Cl, Mg, K, Ca, and P accumulation and the ratio of K^+^/Na^+^ and Na^+^/Cl^−^ are summarized in Table 3.

**TABLE 3:** Variation of major elements in kale leaves (in mg kg^−1^).

G	Mg	P	K	Ca	Na	Cl	K/Na
Control	16,240 ± 110ı***	5051 ± 9 h***	76,350 ± 60 h***	46,020 ± 50e***	17,450 ± 620d***	24,680 ± 20e***	4.38 ± 0.01c***
TYR	18,130 ± 120 h***	5730 ± 10f***	93,880 ± 80f***	48,160 ± 52d***	16,250 ± 670f***	27,820 ± 24d***	5.78 ± 0.01bc***
50‐NaCl	23,190 ± 160e***	6815 ± 12d****	116,500 ± 93d***	40,190 ± 44f***	14,930 ± 620 g***	22,530 ± 18 g***	7.80 ± 0.40b***
100‐NaCl	26,510 ± 150d***	7924 ± 14c**	119,100 ± 92b***	35,200 ± 33 g***	16,660 ± 670e***	23,190 ± 20f***	7.71 ± 0.39b***
200‐NaCl	28,000 ± 160a***	8214 ± 16a***	128,500 ± 95c***	59,040 ± 62b***	21,250 ± 910b***	30,380 ± 20c***	5.61 ± 0.34bc***
TYR‐50	19,030 ± 114 g***	5418 ± 9 g***	92,690 ± 76 g***	49,510 ± 55c***	20,100 ± 880c***	31,610 ± 22b***	4.61 ± 0.01c****
TYR‐100	27,540 ± 154bc***	8034 ± 13b***	102,000 ± 85e***	63,590 ± 66a***	27,000 ± 950a***	36,450 ± 24a***	3.78 ± 0.01 cd***
TYR‐200	21,780 ± 136f***	6547 ± 11e***	132,000 ± 98a****	35,250 ± 35 g***	12,820 ± 540 h***	20,140 ± 18 h***	10.30 ± 0.3a***

*Note:* Mean values (*n* = 3) in the same column for each trait in each group with the same lower‐case letter are not significantly different by Tukey's multiple range test at *p* ≤ 0.05. ***p < 0.001, TYR, tyrosine.

The observed increase in the contents of Mg, K, P, Ca, Na, and Cl, along with a higher K^+^/Na^+^ ratio under 200‐NaCl treatment, suggests a strong ionic response to severe salt stress. Also, the inhibition of Ca, Na, and Cl accumulation by 200‐NaCl (Table 3) has been attributed to the suppression of ion channel activity under high salt concentrations (Sofy et al. 2020). Conversely, the decline in Ca, Na, and Cl levels at lower salinity treatments (50 and 100‐NaCl) indicates that kale can tolerate mild salinity without significant ionic imbalance or toxicity, consistent with findings reported by Arif et al. (2019). Hasana and Miyake (2017) demonstrated a similar pattern in maize under salinity, with increased Ca, Mg, Na, and Cl levels and suppressed K and P accumulation. In another study, Sarker et al. (2018) observed a significant increase in Mg and Na but a decrease in Ca levels in *Amaranthus* cultivars under moderate NaCl, which was coherent with our results. Samec et al. (2021) evidenced that salinity promoted Na accumulation but suppressed K content, leading to a reduced K^+^/Na^+^ ratio in *Brassica* species, with the most substantial decline observed at 200‐NaCl, in contrast to our findings. TYR treatment reduced Na content while enhancing the accumulation of the other five elements, concurrently lowering the Na^+^/Cl^−^ ratio (Table 3), which may be attributed to an important salt tolerance mechanism involving the sequestration of Na^+^ ions into vacuoles mediated by vacuolar Na^+^/H^+^ antiporters (Marium et al. 2021; Zhu et al. 2022). Contrary to our findings, TYR supplementation in unstressed spinach seedlings led to reductions in Mg, P, K, and Ca levels, whereas in Li‐stressed groups, it had no significant impact on these elements except for Ca, which increased (Turfan 2023). Although TYR, in combination with NaCl, broadly enhanced the accumulation of the analyzed elements, its co‐application with 200‐NaCl specifically attenuated the uptake of Ca, Na, and Cl (Table 3). Notably, the K^+^/Na^+^ ratio decreased with TYR‐100, while the Na^+^/Cl^−^ ratio increased at this dose. It was thought that the elevation of Ca, K, Na, and Cl levels in seedlings treated with TYR under 50 and 100‐NaCl conditions indicated that TYR may be more effective in mitigating salt‐induced stress at higher salinity levels (Table 3). It was assumed that the elevation of Ca, K, Na, and Cl levels in seedlings treated with TYR under 50 and 100‐NaCl conditions resulted from TYR's enhanced role in mitigating salt‐induced stress at higher salinity levels. Normally, 200‐NaCl escalates Na^+^ accumulation, disrupting K^+^ uptake by competing for transporters; however, TYR supplementation may counteract this effect by boosting antioxidant defenses and enhancing K^+^ transporter activity in cellular membranes. Under saline conditions, plants accumulate ions such as K^+^, Mg^2+^, and Ca^2+^ to maintain osmotic balance. Mg regulates chlorophyll biosynthesis and enzyme activation, while Mg and Ca strengthen cell walls and facilitate water movement in the apoplast. In this study, the lowest P concentration under control and the highest under 200 mM NaCl may reflect stress‐induced ATP demand (Ciereszko 2018) and oxidative membrane damage, with antagonistic interactions involving K^+^ and Ca^2+^ further facilitating P uptake (Poirier et al. 2022). Corroborating our findings, Sacala et al. (2016) reported increased P content in maize leaves under salt stress, while Turfan (2023) observed a promotive effect of TYR on P accumulation in spinach under alkaline stress. The modulatory effects of amino acids on mineral uptake are also well documented in the literature, with evidence indicating that certain amino acids enhance mineral absorption, whereas others inhibit it. Hussain et al. (2018) highlighted that salt stress increased Na accumulation while reducing Ca and K uptake in *Brassica* cultivars, but the foliar application of proline and glycine betaine mitigated the negative impacts on these mineral levels. Guo et al. (2022) showed that exogenous proline enhanced the K^+^/Na^+^ ratio in alfalfa under salinity by promoting K^+^ uptake, limiting Na^+^ accumulation, and activating APX and SOD activities. Sofy et al. (2020) explored that GB supplementation balanced the K^+^/Na^+^ ratio in bean plants under salt stress by activating antioxidant defenses, preserving membrane integrity, and restricting Na^+^ uptake. Differences in kale's responses to NaCl concentrations may be related to the duration and frequency of NaCl application, the type of salt used, the plant's genotype, and antagonistic interactions between elements. Alasvandyaria and Mahdavia (2018) observed that the antagonistic interaction between Na and K under NaCl stress increased Na accumulation in safflower, whereas foliar application of GB reduced Na content and elevated Ca levels. Furthermore, antagonistic interactions among P, K, and Ca have been documented, with elevated levels of Mg^2+^, K^+^, or Ca^2+^ adversely affecting the absorption of the others due to competition at shared transport sites (Zhu et al. 2022).

## 4 Conclusions

This study demonstrated that foliar application of TYR in Kale under varying salt stress levels (50, 100, and 200 mM) triggered distinct morphological, physiological, and biochemical responses. TYR alone significantly enhanced shoot and root growth, leaf size, and fresh weight compared to the control, while these parameters were most suppressed under 200 mM NaCl. TYR supplementation under NaCl stress partially improved growth and stress‐related metrics. Moderate NaCl levels (50–100 mM) elevated Chl‐b, total chlorophyll, lutein, TPC, anthocyanin, proline, K/Na ratio, POD‐CAT activity, and reduced MDA and H_2_O_2_. TYR combined with NaCl modulated ion accumulation, notably enhancing the K and K/Na ratio at high salinity (200‐mM), while reducing Ca, Na, Cl, and Na/K. Overall, TYR‐50 induced mild improvement, TYR‐100 showed suppression, and TYR‐200 reinstated positive trends, indicating that higher TYR doses may counteract severe salinity effects. These findings support TYR as a promising biostimulant for salt stress mitigation, warranting further investigation into its molecular mechanisms and broader agricultural potential.

## Author Contributions

**Nezahat Turfan:** conceptualization (equal), investigation (equal), resources (equal), supervision (lead), writing – original draft (lead), writing – review and editing (lead). **İskender Khubalıyev:** investigation (supporting), methodology (equal). **Kübra Tekşen:** data curation (equal), investigation (equal), software (equal), visualization (lead), writing – review and editing (equal). **Ergin Murat Altuner:** conceptualization (equal), data curation (equal), writing – review and editing (equal).

## Conflicts of Interest

The authors declare no conflicts of interest.

## Data Availability

The data that support the findings of this study are available on request from the corresponding author. The data are not publicly available due to privacy or ethical restrictions.
