# Correction to “Investigating Exogenous Tyrosine Supplements on the Responses of the Kale Plant to Salinity Stress”

**DOI:** 10.1002/fsn3.70873

**Published:** 2025-08-27

Turfan, N., İ. Khubalıyev, K. Tekşen, and E. M. Altuner “Investigating Exogenous Tyrosine Supplements on the Responses of the Kale Plant to Salinity Stress.” *Food Science & Nutrition*, vol. 13, no. 7, 2025, p. e70660, https://doi.org/10.1002/fsn3.70660

In the originally published article, Figure 6 was incorrect. The correct figure appears below:

**FIGURE 6:**
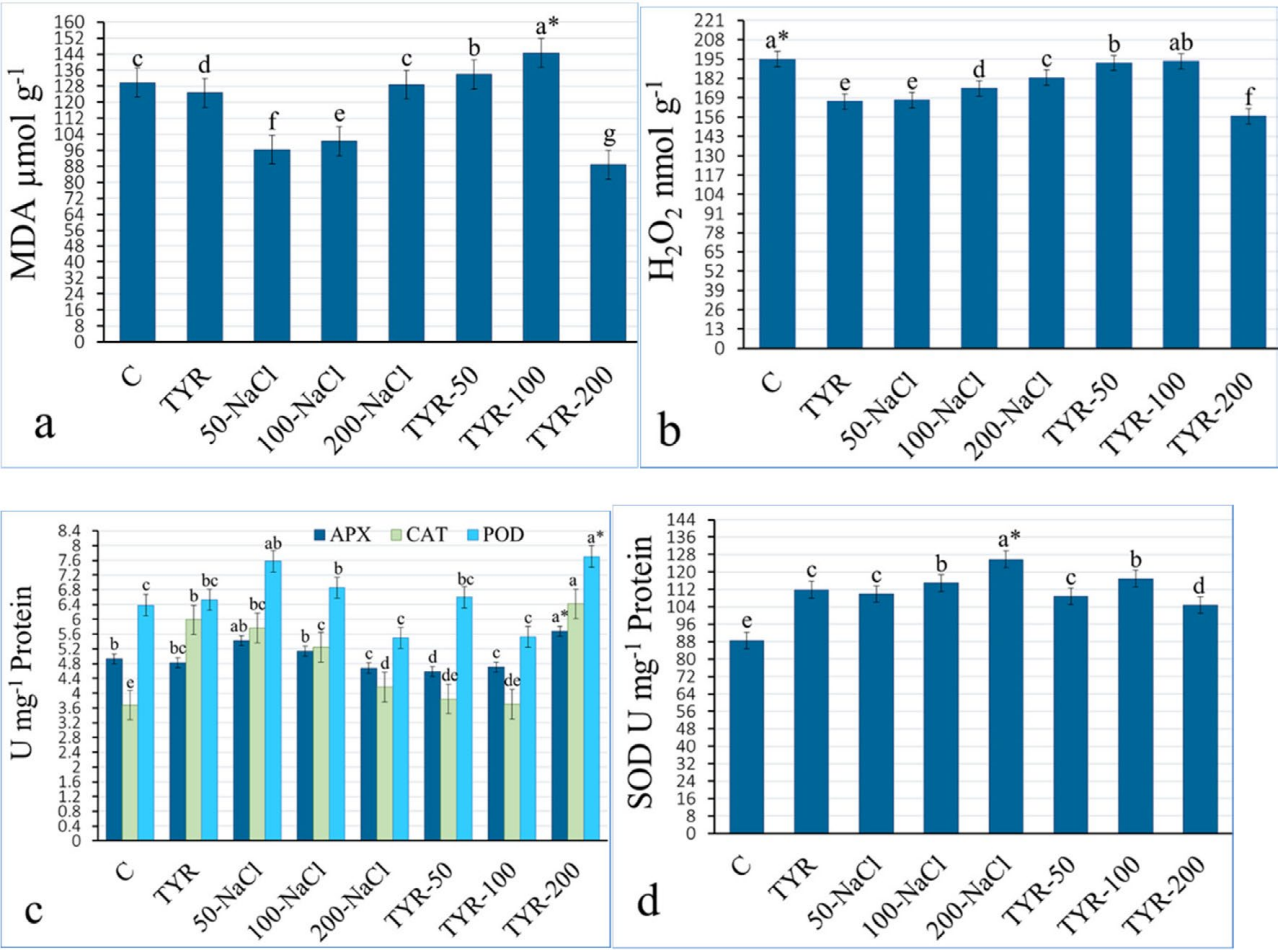
Variation of (a) malondialdehyde (MDA), (b) hydrogen peroxide (H_2_O_2_) concentrations, and the activity of (c) APX, CAT, POD, and (d) SOD enzymes in the kale leaves. *Mean values (*n* = 3) in the same column for each trait in each group with the same lower‐case letter are not significantly different by Tukey's multiple range test at *p* ≤ 0.05. TYR, Tyrosine.

We apologize for this error.

